# Predictors of cognitive impairment in patients undergoing ileostomy for colorectal cancer: a retrospective analysis

**DOI:** 10.7717/peerj.15405

**Published:** 2023-06-05

**Authors:** Jing Xu, Yuelan Yang, Die Hu

**Affiliations:** 1 Department of Gastroenterology, Changxing People’s Hospital, Changxing, China; 2 Department of Rehabilitation Medicine, Changxing People’s Hospital, Changxing, China; 3 Department of Ultrasound Medicine, Changxing People’s Hospital, Changxing, China

**Keywords:** Postoperative, Cognitive impairment, Retrospective analysis, Clinical predictive models, Ileostomy

## Abstract

**Background:**

Early detection of cognitive impairment in patients undergoing ileostomy for colorectal cancer may help improve patient outcomes and quality of life. Identifying risk factors and clinically accessible factors is crucial for prevention and treatment.

**Objective:**

This retrospective study aimed to identify risk factors for post-operative cognitive impairment in patients undergoing ileostomy for colorectal cancer and to explore potential factors for its prevention and treatment.

**Methods:**

A total of 108 cases were selected and included in the study. Patient data including general characteristics, disease stage, complications, and chemotherapy status were collected, and sleep quality and cognitive function were assessed using questionnaires and follow-up. Patients were randomly divided into training and validation groups. A random forest model was used to rank clinical features based on their contribution to predicting the prognosis of cancer-related cognitive impairment (CRCI). Nomograms were constructed using the support vector machine–recursive feature elimination (SVM-RFE) method, and the minimal root-mean-square error (RMSE) values were compared to select the best model. Regression analysis was performed to determine independent predictors.

**Results:**

Significant differences were observed in age, body mass index (BMI), alcohol consumption, frequency of physical activity, comorbidity, and cancer-related anemia (CRA) between the CRCI and non-CRCI groups. Random forest analysis revealed that age, BMI, exercise intensity, PSQI scores, and history of hypertension were the most significant predictors of outcome. Univariate logistic regression analysis of 18 variables revealed that age, alcohol consumption, exercise intensity, BMI, and comorbidity were significantly associated with the outcome of CRCI (*p* < 0.05). Univariate and multivariate models with P-values less than 0.1 and 0.2, respectively, showed better predictive performance for CRCI. The results of univariate analysis were plotted on a nomogram to evaluate the risk of developing CRCI after colorectal cancer surgery. The nomogram was found to have good predictive performance. Finally, regression analysis revealed that age, exercise intensity, BMI, comorbidity, and CRA were independent predictors of CRCI.

**Conclusions:**

This retrospective cohort study revealed that age, exercise intensity, BMI, comorbidity, CRA, and mobility are independent predictors of cognitive impairment in patients undergoing ileostomy for colorectal cancer. Identifying these factors and potential factors may have clinical implications in predicting and managing post-operative cognitive impairment in this patient population.

## Introduction

Cognitive impairment encompasses a multifaceted and intricate array of cognitive domains, including executive function, learning and memory, perceptual-motor function, language, complex attention, and social cognition (Alzheimer’s Association, 2015). Among elderly Chinese adults, cognitive decline prevails at 15.4%, significantly impacting their quality of life (Deng et al., 2021). Tumor-bearing individuals exhibit heightened susceptibility to cognitive impairments with advancing age (Lange et al., 2019). Research has demonstrated that early identification of neurovascular markers for tumor-induced cognitive decline in clinical environments may thwart the progression of tumor-induced neurodegeneration (Sales et al., 2019; Lukiw et al., 2020). Consequently, early interventions targeting cognitive decline can impede the onset and progression of neurovascular disorders such as Alzheimer’s disease.

Colorectal cancer, a chronic debilitating ailment, frequently presents with cognitive decline (Cruzado et al., 2014; Huehnchen et al., 2020; Kim et al., 2021). In 2020, an estimated 2.3 million new cancer cases and nearly 1.8 million cancer-related fatalities will occur globally, with colorectal cancer constituting approximately 10% of all new cancer diagnoses and ranking as the second leading cause of cancer-related deaths (Sung et al., 2021). Investigating cognitive decline in elderly patients with limited samples, particularly those undergoing post-chemotherapy colorectal cancer surgery, may yield valuable insights (Oh & Kim, 2016). Predictive models for ascertaining the risk of cancer-induced cognitive decline following chemotherapy were developed based on age, body mass index, colostomy, comorbidities, CRA, depression, diabetes, QLQ-C30 score, exercise, hypercholesterolemia, diet, marital status, education, and pathological stage in colorectal cancer patients (Zhou et al., 2021). Ileostomy, a prevalent surgical intervention for colorectal cancer patients with bowel obstructions, seeks to alleviate bowel pressure and enhance patients’ quality of life (Veld et al., 2020). Two-thirds of stage III colorectal cancer patients (and some high-risk stage II patients) undergo adjuvant chemotherapy (Kannarkatt et al., 2017). However, ileostomy and other treatments, such as chemotherapy, can trigger cognitive decline in patients due to treatment-related complications, cancer-related anemia (CRA), and other factors (Andreev et al., 2020). While prior research has explored the association between ileostomy for colorectal cancer and cognitive decline, as well as briefly examining certain risk factors, the explicit factors predisposing patients to cognitive decline following ileostomy for colorectal cancer remain ambiguous. Moreover, no predictive models or clinical trials have been undertaken to explicate this relationship.

Predictive models can also assist physicians in implementing precision medicine, thereby formulating more personalized and efficacious treatment plans (Pencina & Peterson, 2016; Obermeyer & Emanuel, 2016; Kang et al., 2020; Wu et al., 2021; Lin et al., 2022; Chen et al., 2022a). These models can enhance research efficiency, accelerate the development of novel pharmaceuticals, and optimize therapeutic strategies, ultimately making significant contributions to the improvement of human health. This study aimed to identify clinically accessible neurovascular-related factors for disease prevention and treatment. Accordingly, we sought to collect and analyze clinical data in this retrospective analysis to discern factors associated with ileostomy for colorectal cancer-induced cognitive decline. This information will furnish clinical and nursing care with invaluable factors for disease prevention and treatment.

## Materials & Methods

### Study design and participants

**Figure 1:**
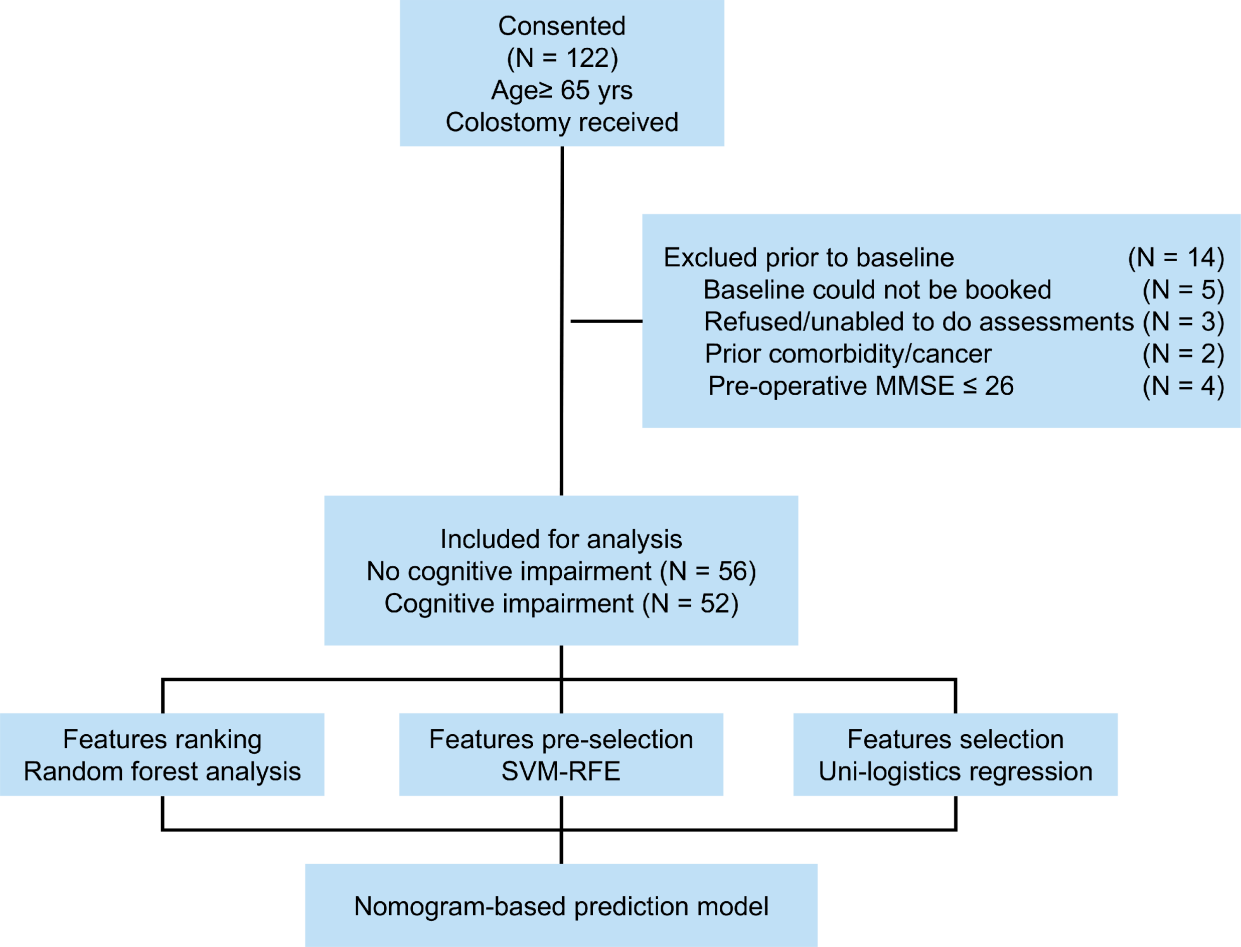
Flow diagram of this study.

Between September 2018 and September 2021, 122 patients aged 65 or older underwent colorectal cancer ileostomy procedures at Changxing People’s Hospital. Patient information was collected from medical records during hospitalization, with subsequent telephone appointments and community follow-ups. Inclusion criteria included postoperative pathologically confirmed colorectal cancer, age of 65 years, and colorectal cancer fistula. Exclusion criteria were as follows: (1) patients with metastatic disease, emergency surgery, or a life expectancy less than 6 months, (2) refusal to participate or inability to assess (*N* = 3), (3) lack of baseline information(including clinical or pathological data), death during the study, (4) concomitant tumors or diseases (*N* = 2), (5) unexplained fever or infection, and (6) history of psychotropic substance use. The study’s flow structure is illustrated in Fig. 1. Informed consent was obtained from the patient or family after the study was approved by the hospital ethics committee. Finally, 108 cases were selected and included in the study. This study was approved by the Institutional Ethics Review Board of Changxing People’s Hospital (approval No. 2021-033). Informed written consent was obtained from all the patients.

### Clinical feature data collection

Clinical histories included general patient characteristics (age, BMI, gender, home address, education, marital status, smoking, and alcohol consumption) and medical history (hypertension, hyperlipidemia, and diabetes). Surgery-related data determined the cancer stage, complications, and chemotherapy. The World Health Organization and American Cancer Institute (NCI) anemia grading scales define tumor-associated anemia (CRA) during hospitalization as hemoglobin (Hb) > 120 g/L in men and > 110 g/L in women. Telephone and community follow-ups provided comprehensive records of the patient’s diet, exercise intensity, and mobility. The three mobility types are independent mobility without aid, independent mobility with a walking aid, and independent mobility with an assistive device (15). The Pittsburgh Sleep Quality Index (PSQI) evaluates patients’ sleep quality (scores range from 0 to 21) (Buysse et al., 1989; Zhou et al., 2021).

### Cognitive function assessment

Cognitive function was assessed using the Montreal Cognitive Assessment (MoCA) (Nasreddine et al., 2005) and the Functional Assessment of Cancer Therapy-Cognitive Function (FACT-Cog) (Cheung & Chan, 2013). All patients were tested by experienced clinical neuropsychologists and completed corresponding patient-reported outcome questionnaires at the final follow-up visit for further diagnosis of tumor-related cognitive impairment (CRCI) (Zhou et al., 2021).

### Machine learning and feature filtering

Patients were randomly assigned to training and validation groups(group ratio 7:3). The random forest model quantifies the importance of each feature and balances error in high-dimensional feature data with unbalanced data (Buxton et al., 2018). As previous researches, a random forest model was applied to explore the contribution of each clinical feature to the CRCI outcome. Support Vector Machine-Recursive Feature Elimination (SVM-RFE) was employed to model and filter patient variable characteristics (Huang et al., 2014; Ying et al., 2021). Support vector machines (SVMs) are effective at removing redundant features and demonstrate high accuracy in classifying small sample datasets. The Root Mean Squared Error (RMSE) metric is applied to filter models, and variables required for the best model are determined based on the minimum RMSE value.

### Statistical analysis

Count data were expressed as percentages, and chi-square tests were used to determine the statistical differences between the two groups. Means and standard deviations of age, BMI, and PSQI were calculated. The significance of continuous variables between the two groups was determined by Student’s *t*-test. Logistic regression analysis was employed for univariate and multivariate analyses to identify risk factors. Results were presented as odds ratios (OR) with a 95 percent confidence interval (95% CI). In constructing clinical prediction models, variables with *P* < 0.2 and *P* < 0.1 in the univariate analysis were incorporated into multivariate analyses for variable screening. Variables with *P* < 0.2 in the multifactorial analysis were used in the construction of the CRCI prediction model. Model accuracy was assessed using ROC curves to visualize AUC values. Calibration curves and C-indices were employed to evaluate the predictive power of models with high AUC values. To assess the clinical applicability of the models, DCA curves were used. R software (version 3.6.3) was utilized for all statistical analyses. A *P* value less than 0.05 was considered statistically significant.

## Results

### Patients and clinical characteristics

A total of 122 patients were enrolled in the study, and 108 were included for analysis. Among the included patients, 57.4% were female, and the mean age was 72.8 years (range 65-89 years). Baseline characteristics of the included patients are presented in Table 1. Significant differences were observed in age (*P* < 0.01), BMI (*P* < 0.01), and alcohol consumption (*P* = 0.037) between the CRCI and non-CRCI groups.

**Table 1:** General characteristics of patients.

		CRCI		Total	*χ*2	*p*
		No (*n* = 56)	Yes (*n* = 52)	(*n* = 108)		
Age		70.93 ± 5.24	74.75 ± 4.84	72.77 ± 5.38	−3.93	<0.01^**^
BMI		25.37 ± 3.37	23.74 ± 2.97	24.58 ± 3.27	2.652	<0.01^**^
Gender						
	Female	35 (62.50)	27 (51.92)	62 (57.41)	1.234	0.267
	Male	21 (37.50)	25 (48.08)	46 (42.59)		
Address						
	Centra	12 (21.43)	13 (25.00)	25 (23.15)	0.429	0.807
	Rural	33 (58.93)	31 (59.62)	64 (59.26)		
	Urban	11 (19.64)	8 (15.38)	19 (17.59)		
Education						
	Elementary	18 (32.14)	21 (40.38)	39 (36.11)	1.388	0.5
	Illiteracy	1 (1.79)	2 (3.85)	3 (2.78)		
	Secondary or above	37 (66.07)	29 (55.77)	66 (61.11)		
Marital_status						
	Married	39 (69.64)	44 (84.62)	83 (76.85)	4.016	0.134
	Single or divorced	2 (3.57)	2 (3.85)	4 (3.70)		
	Widowed	15 (26.79)	6 (11.54)	21 (19.44)		
Hypertension						
	No	44 (78.57)	34 (65.38)	78 (72.22)	2.337	0.126
	Yes	12 (21.43)	18 (34.62)	30 (27.78)		
Dyslipidemia						
	No	45 (80.36)	41 (78.85)	86 (79.63)	0.038	0.846
	Yes	11 (19.64)	11 (21.15)	22 (20.37)		
Diabetes						
	No	46 (82.14)	40 (76.92)	86 (79.63)	0.453	0.501
	Yes	10 (17.86)	12 (23.08)	22 (20.37)		
Smoking						
	No	46 (82.14)	41 (78.85)	87 (80.56)	0.187	0.665
	Yes	10 (17.86)	11 (21.15)	21 (19.44)		
Drinking						
	No	37 (66.07)	24 (46.15)	61 (56.48)	4.352	0.037^*^
	Yes	19 (33.93)	28 (53.85)	47 (43.52)		

**Notes.**

* *p* < 0.05

** *p* < 0.01

Table 2 presents information on surgical treatment and follow-up of patients. The overall incidence of surgery-related complications was 24%. Significant differences were observed in exercise intensity, complications, and the presence of CRA between the CRCI and non-CRCI groups.

**Table 2:** Surgery- and survival-related characteristics.

		CRCI		Total	*χ*2	*p*
		No (*n* = 56)	Yes (*n* = 52)	(*n* = 108)		
PSQI		6.57 ± 3.81	7.52 ± 3.45		−1.35	0.18
Exercise_strength						
	High	17 (30.36)	6 (11.54)	23 (21.30)	10.65	0.005^**^
	Low	16 (28.57)	30 (57.69)	46 (42.59)		
	Medium	23 (41.07)	16 (30.77)	39 (36.11)		
Pathological_stage						
	III	25 (44.64)	24 (46.15)	49 (45.37)	0.025	0.875
	I–II	31 (55.36)	28 (53.85)	59 (54.63)		
Diet						
	Balanced diet	15 (26.79)	12 (23.08)	27 (25.00)	0.22	0.896
	Carnivorous	26 (46.43)	26 (50.00)	52 (48.15)		
	Vegetarian	15 (26.79)	14 (26.92)	29 (26.85)		
Complications						
	No	50 (89.29)	34 (65.38)	84 (77.78)	8.912	0.003^**^
	Yes	6 (10.71)	18 (34.62)	24 (22.22)		
CRA						
	No	17 (30.36)	4 (7.69)	21 (19.44)	8.843	0.003^**^
	Yes	39 (69.64)	48 (92.31)	87 (80.56)		
Chemotherapy						
	No	42 (75.00)	45 (86.54)	87 (80.56)	2.292	0.13
	Yes	14 (25.00)	7 (13.46)	21 (19.44)		
Mobility						
	Dependent of support care or unable to move	2 (3.57)	5 (9.62)	7 (6.48)	5.083	0.079
	Independent	33 (58.93)	20 (38.46)	53 (49.07)		
	Independent with walking aid	21 (37.50)	27 (51.92)	48 (44.44)		

**Notes.**

* *p* < 0.05

** *p* < 0.01

### Screening and ranking of important clinical features

**Figure 2:**
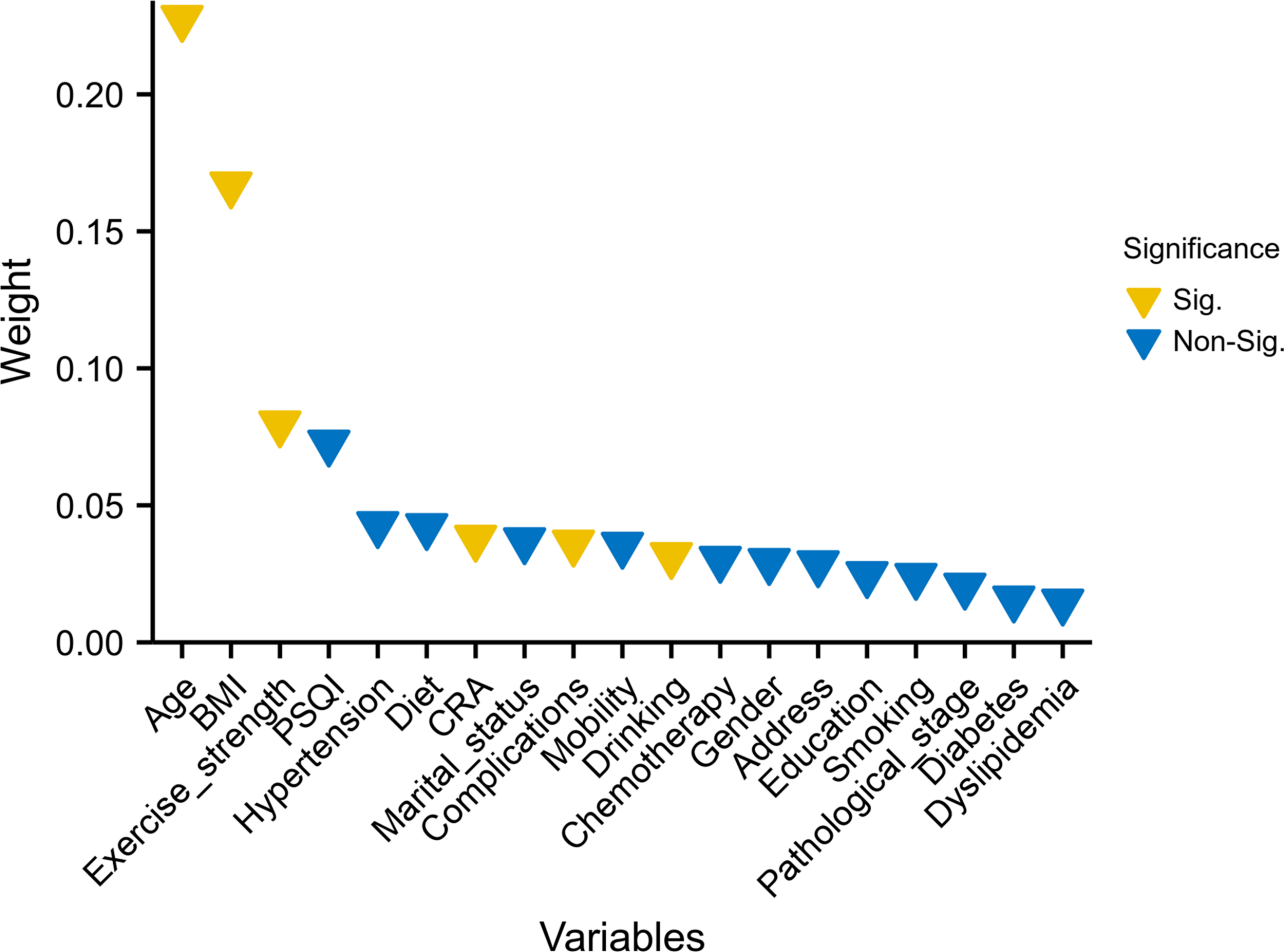
Random forest model ranked the 19 variables based on their contribution to predicting the outcome. Variables that differed between the groups are highlighted in yellow.

**Figure 3:**
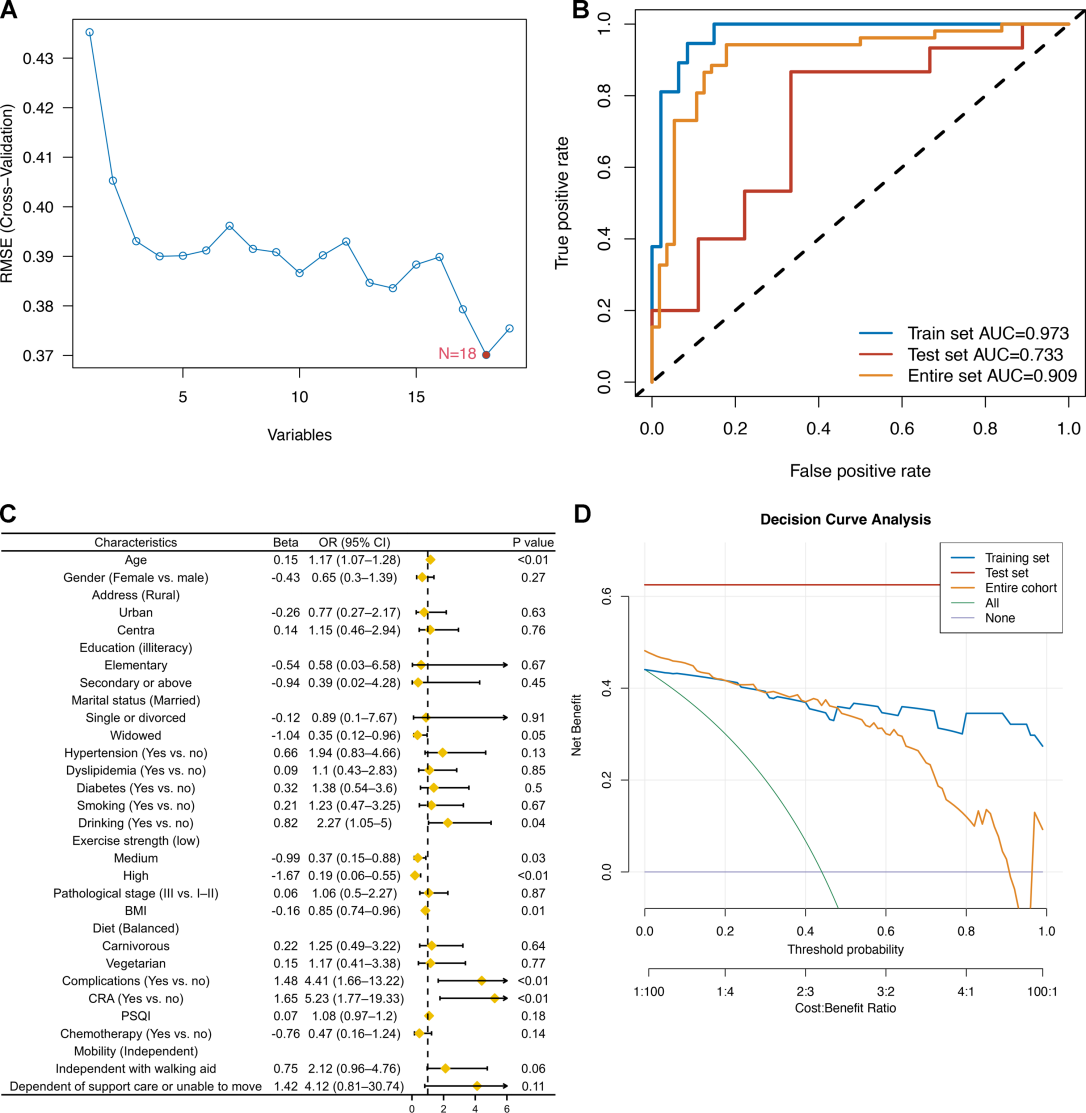
Construction of an SVM-RFE based outcome prediction model. (A) Models were constructed using 18 variables relevant for outcome prediction according to SVM-RFE; (B) for the training set, test set, and overall set, SVM models constructed based on filtered variables performed well; (C) these 18 screened variables showed good predictive power for outcome based on univariate logistic regression analysis; (D) clinical application of the SVM model was determined by a decision curve analysis.

The ranking of characteristic variables based on their contribution to predicting the outcome of CRCI was determined using a random forest model. The top five predictors of the outcome of CRCI were age, BMI, exercise intensity, PSQI scores, and history of hypertension, as shown in Fig. 2. The construction of the SVM-RFE-based clinical predictive model and the variable screening process are demonstrated in Fig. 3. The SVM model was used to screen 18 variables, and the AUC value for the overall cohort was 0.909 (Figs. 3A–3B). These 18 variables performed well in the one-way logistic regression model (Fig. 3C). The outcomes of CRCI were significantly associated with age, alcohol consumption, exercise intensity, BMI, and comorbidity (*P* < 0.05). The clinical applicability and safety of the model were demonstrated using DCA (Fig. 3D). Overfitting in the machine learning process resulted in higher AUC values of the randomised Senri and SVM models in the training set compared to the validation set.

### Logistic regression model for predicting cognitive impairment

Two models were constructed based on pre-set filters in univariate analysis (Table 3). Model 1 incorporated age, exercise intensity, BMI, comorbidity, CRA, and PSQI scores, while Model 2 included age, exercise intensity, BMI, comorbidity, CRA, and mobility. Both models were used to predict CRCI, and Table 3 presents the coefficients and statistical test results of each variable.

### Modelling and construction of nomograms

**Figure 4:**
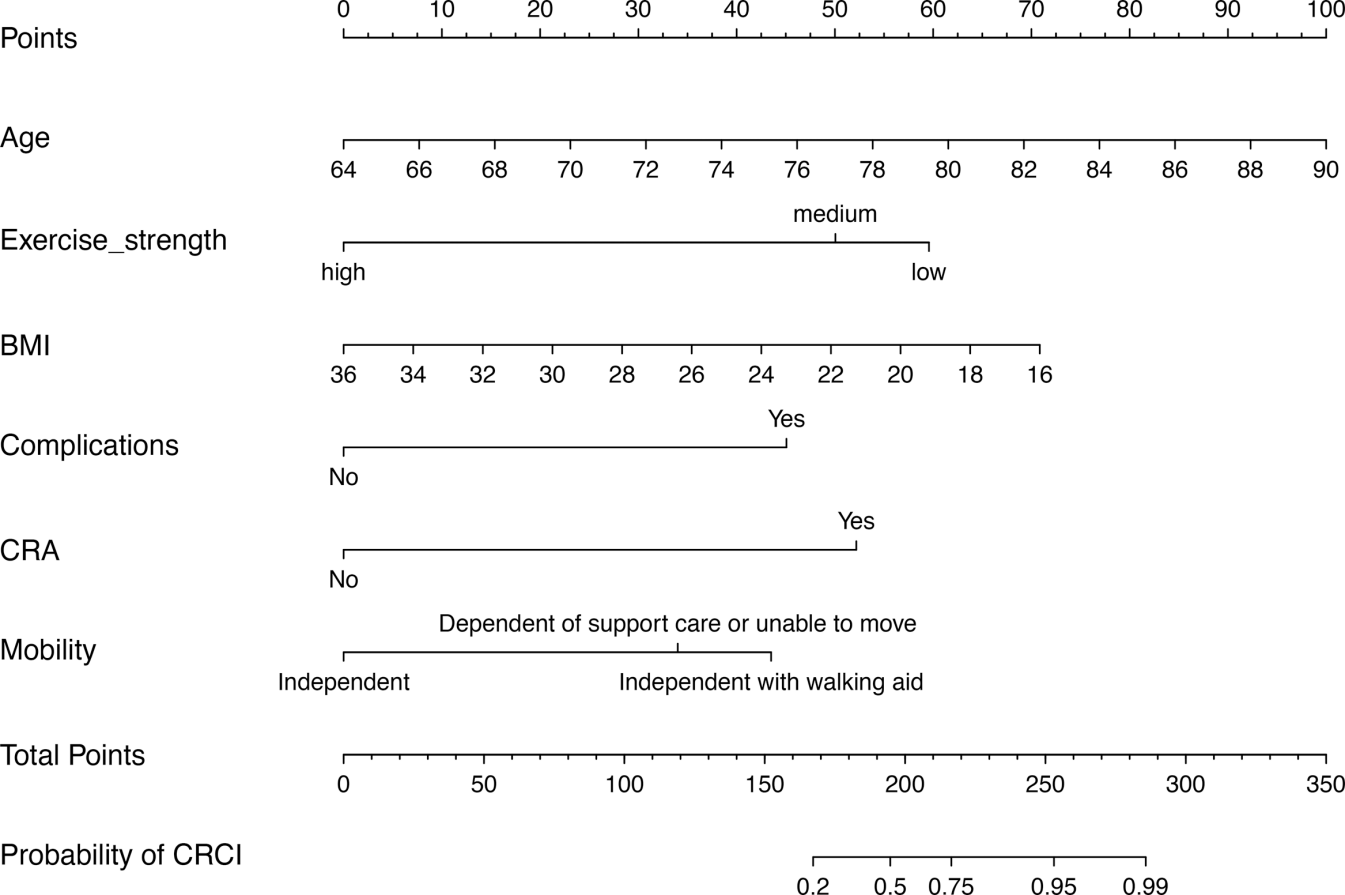
Construction of an SVM-RFE based outcome prediction model. (A) Models were constructed using 18 variables relevant for outcome prediction according to SVM-RFE. (B) For the training set, test set, and overall set, SVM models constructed based on filtered variables performed well. (C) These 18 screened variables showed good predictive power for outcome based on univariate logistic regression analysis. (D) Clinical application of the SVM model was determined by a decision curve analysis.

**Figure 5:**
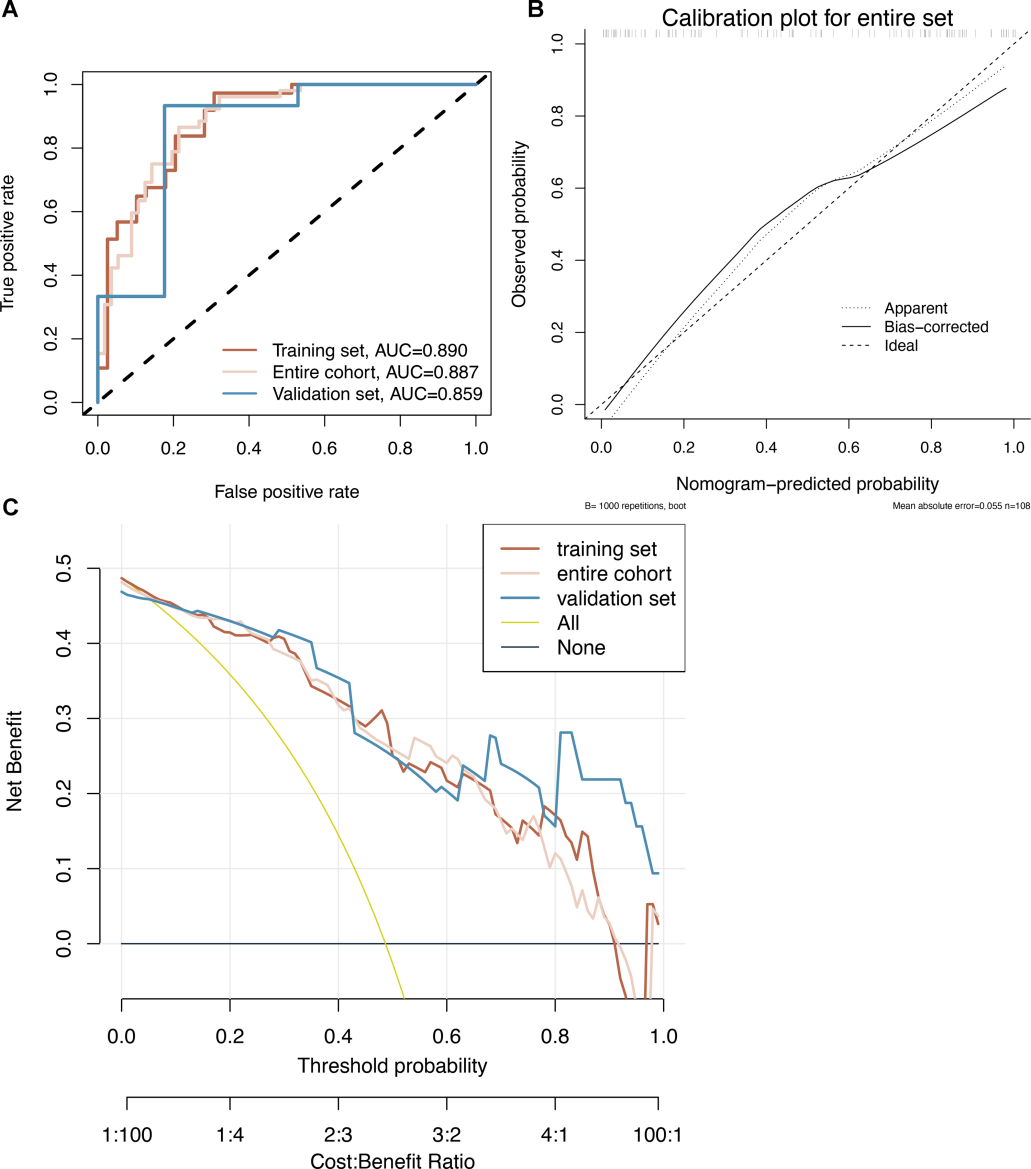
(A–C) Nomogram prediction model. CRCI risk can be assessed by using a nomogram prediction model based on multifactorial logistics.

Model 2 was used to develop an easy-to-use clinical tool to evaluate the risk of developing CRCI after the formation of a colorectal fistula, as shown in Fig. 4. The risk of cognitive impairment can be evaluated by calculating the score of patients for each entry, thus guiding interventions in clinical settings. The multi-factor logistic regression-based clinical predictive model was validated using the nomogram, demonstrating good agreement between the predicted and actual results (Fig. 5B). DCA plots indicated that the model had good clinical applicability and safety (Fig. 5C). These results suggest that the model can efficiently predict the outcomes of CRCI.

### Independent predictors of CRCI

**Figure 6:**
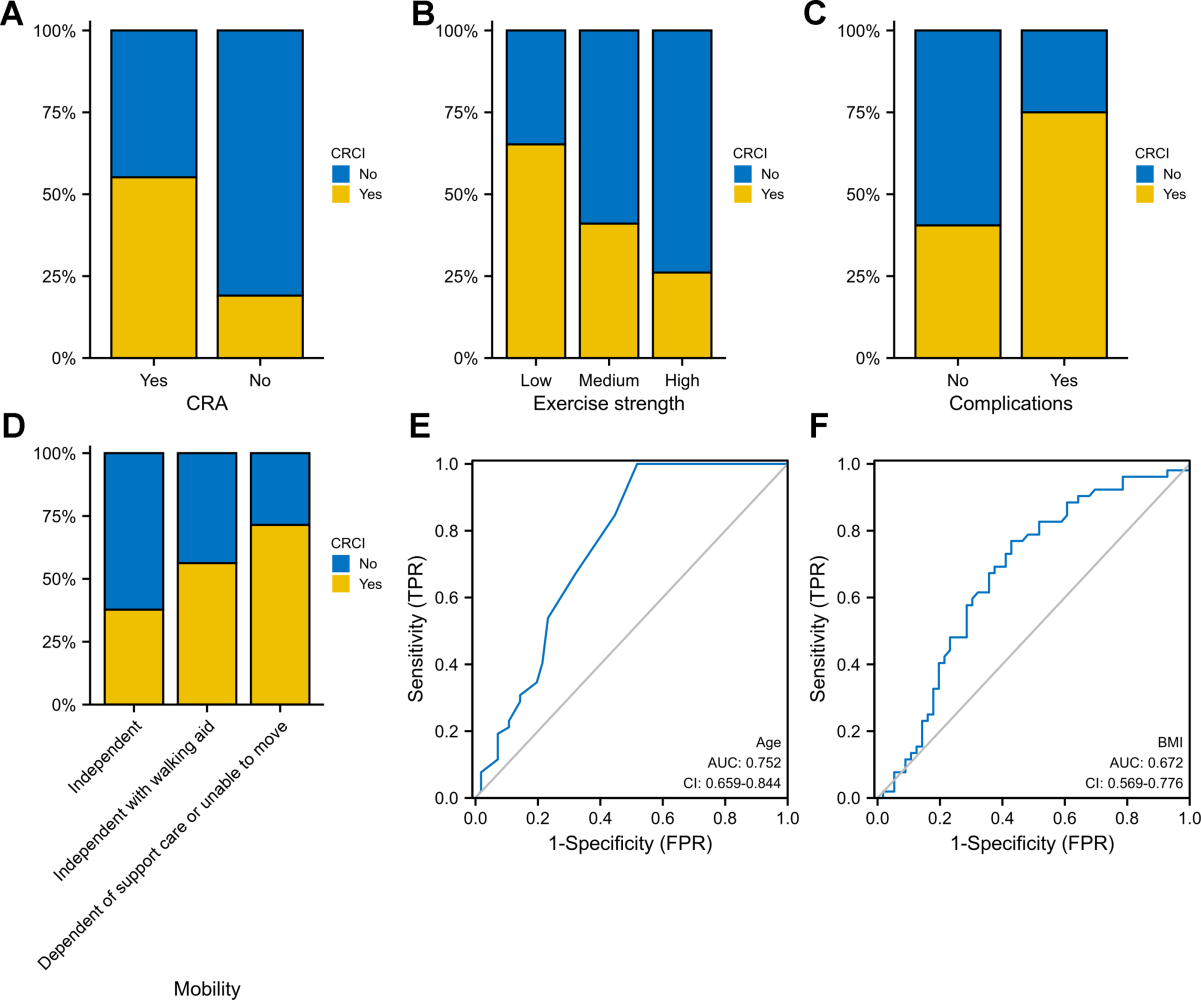
Evaluation of a multifactor logistic regression model for CRCI prediction. (A) ROC analysis shows that the constructed model performs well on the training, test, and overall data sets. (B) Model predictions and true results are well aligned on the calibration curve. (C) The decision curve indicates that the model is clinically applicable.

Age, exercise intensity, BMI, comorbidity and CRA were identified as independent predictors of CRCI in both univariate and multivariate logistic regression analyses (*P* < 0.05). CRCI development was associated with advanced age (OR = 1.17; 95% CI [1.07–1.28]), comorbidity (OR = 4.41; 95% CI [1.66–13.22]) and CRA (OR = 5.23; 95% CI [1.77–19.33]) (Table 4). Higher BMI had protective effects against CRCI development (OR = 0.85; 95% CI [0.74–0.96]). Compared with low-intensity exercise, high-to-moderate-intensity exercise had better protective effects against CRCI development. CRCI was more prevalent in the CRA group than in the non-CRA group (Fig. 6A). The high-intensity-exercise group had the lowest incidence of CRCI (Fig. 6B). The incidence of CRCI was higher among patients with surgery-related complications than among those without surgery-related complications (Fig. 6C). The independent-mobility group had the lowest incidence of CRCI; however, the results were not significant (Fig. 6D). Age (AUC = 0.752) and BMI (AUC = 0.672) were good predictors of CRCI (Figs. 6E–6F).

**Table 3:** Multivariable logistics regression models to explore the CRCI-related factors.

Model	Factor	Beta	OR (95% CI)	*P*
Model 1				
	Age	0.17	1.19 (1.07–1.34)	<0.01
	Exercise strength (low vs. high)	1.79	5.98 (1.41–30.37)	0.02
	Exercise strength (medium vs. high)	1	2.71 (0.64–13.43)	0.19
	BMI	−0.21	0.81 (0.68–0.94)	<0.01
	Complications (Yes vs. no)	2.13	8.42 (2.32–38.69)	<0.01
	CRA (Yes vs. no)	2.21	9.08 (2.26–50.14)	<0.01
	PSQI	0.12	1.13 (0.99–1.31)	0.08
Model 2				
	Age	0.19	1.21 (1.09–1.38)	<0.01
	Exercise strength (low vs. high)	3.01	20.24 (4.18–123.8)	<0.01
	Exercise strength (medium vs. high)	2.53	12.51 (2.34–84.52)	<0.01
	BMI	−0.18	0.84 (0.71–0.98)	0.03
	Complications (Yes vs. no)	2.28	9.73 (2.44–50.38)	<0.01
	CRA (Yes vs. no)	2.63	13.93 (2.74–105.31)	<0.01
	Mobility (Independent vs. dependent^a^)	−1.72	0.18 (0.01–1.7)	0.15
	Mobility (Independent with walking aid vs. dependent^a^)	0.48	1.62 (0.14–15.89)	0.68

**Notes.**

a Dependent of support care or unable to move.

**Table 4:** Results of univariate and multivariate logistic regression analyses.

	Univariable				Multivariable			
Variable	Beta	OR (95% CI)	*P*		beta	OR (95% CI)	*P*
Age	0.15	1.17 (1.07–1.28)	<0.01		0.16	1.17 (1.06–1.32)	<0.01
Drinking (yes versus no)	0.82	2.27 (1.05–5)	0.04		0.68	1.98 (0.72–5.61)	0.19
Exercise strength							
Medium versus low	−0.99	0.37 (0.15–0.88)	0.03	Low *vs.* high	1.87	6.51 (1.54–32.51)	0.01
High versus low	−1.67	0.19 (0.06–0.55)	<0.01	Medium *vs.* high	1.2	3.31 (0.78–16.64)	0.12
BMI	−0.16	0.85 (0.74–0.96)	0.01		−0.18	0.83 (0.71–0.97)	0.02
Complications (yes versus no)	1.48	4.41 (1.66–13.22)	<0.01		2	7.4 (2–34.89)	<0.01
CRA (yes versus no)	1.65	5.23 (1.77–19.33)	<0.01		2.15	8.6 (2.09–49.61)	<0.01

## Discussion

A considerable fraction of cancer patients undergo cognitive deterioration and functional decline, particularly among individuals aged 85 and above (DeSantis et al., 2019). Consequently, early recognition of cognitive impairment in cancer patients might bolster their chances of survival. In this retrospective cohort investigation, we scrutinized risk factors for post-operative cognitive impairment in patients receiving ileostomy for colorectal cancer. We discovered that age, exercise intensity, BMI, comorbidity, CRA, and mobility were independent predictors of cognitive impairment within this patient demographic.

Cognitive impairment and its correlation with colorectal cancer have garnered increasing interest in recent years, with patients exhibiting a higher prevalence of cognitive impairment compared to the general population (Vardy, Rourke & Tannock, 2007; Janelsins et al., 2014). Factors such as tumor-induced systemic inflammation, oxidative stress, and pro-inflammatory cytokines, along with cancer treatments like chemotherapy and radiotherapy, may contribute to cognitive decline (Janelsins et al., 2014). Although evidence is limited, ileostomy for colorectal cancer has been suggested to indirectly influence cognitive function through postoperative complications like dehydration, electrolyte imbalance, and infection, as well as psychological stressors, such as body image concerns and reduced quality of life (Tan et al., 2008; Hanna, Vinci & Pigazzi, 2015; Paulson et al., 2022). Further research is needed to better understand the underlying biological mechanisms and develop targeted interventions to prevent or mitigate cognitive decline in this patient population.

To construct a clinical prediction model for CRCI outcomes, we analyzed the clinical information of patients, incorporating variables such as age, exercise intensity, BMI, comorbidities, CRA, and mobility. Alzheimer’s disease and cancer frequently coexist in the elderly population, with CRCI showing a significant correlation with age. The likelihood of cognitive impairment increases with age (Baiano et al., 2020) and may stem from the burden of cancer and its subsequent treatment (Snaedal, 2018). The association between age and cognitive decline is well-established, and it is known that the risk of cognitive impairment increases with age (Lenehan et al., 2015; Legdeur et al., 2018). Studies have indicated that older Chinese individuals with higher BMI and overweight status exhibit a reduced risk of cognitive impairment (Dos Santos et al., 2018). Additionally, our findings underscore the importance of CRA, BMI, and exercise intensity as predictors of cognitive impairment in patients undergoing ileostomy for colorectal cancer. Anemia not only impairs cognitive function, growth, and physical capability but also serves as a crucial determinant of health (Van der Harst et al., 2012). Physical activity also improved cognition in people with mild cognitive impairment or dementia, according to a meta-analysis (Karssemeijer et al., 2017). According to the clinical prediction model, age, exercise intensity, BMI, comorbidities, and CRA are independent predictors of CRCI, as depicted in a nomogram.

The discernment of these risk factors and potential biomarkers bears clinical ramifications for the prognostication and management of postoperative cognitive impairment in patients undergoing ileostomy for colorectal cancer. Early detection methods and intervention for cognitive decline may contribute to delaying the onset and progression of neurovascular disorders such as Alzheimer’s disease (Lenehan et al., 2015; Chen et al., 2022c; Chen et al., 2022b; Du et al., 2022). Moreover, our findings may facilitate the development of personalized treatment and care plans for these patients, taking into consideration their individual risk factors for cognitive impairment. This study exhibits certain limitations, including its retrospective design, which may have engendered selection bias. Additionally, the sample size was comparatively small, and further studies with larger sample sizes and prospective designs are imperative to substantiate our findings. While the model employs a validation set of extant follow-up patients with pertinent clinical data incorporated into the prediction model, independent datasets or prospective studies are absent for model validation. Notwithstanding these limitations, this study has identified numerous factors contributing to the emergence of CRCI following colorectal ileostomy. We have devised an easily interpretable nomogram that enables early identification of patients’ risk factors for developing CRCI, laying the groundwork for timely prevention or intervention measures. Our study also imparts valuable insights into the risk factors for cognitive impairment in patients undergoing ileostomy for colorectal cancer and underscores the necessity for continued research in this domain. In subsequent applications, we aspire to further optimize the design and validate it using an independent validation set, in addition to scrutinizing a larger cohort.

## Conclusion

In this study, we constructed a nomogram-based clinical predictive model employing age, exercise intensity, BMI, comorbidities, and CRA to assess the risk of CRCI following colorectal ileostomy formation. This model provides theoretical support for thwarting neurovascular diseases in clinical settings and may augment survival following surgical treatment for colorectal cancer. However, to confirm its predictive accuracy, additional validation studies with larger sample sizes and independent datasets are required.

##  Supplemental Information

10.7717/peerj.15405/supp-1 Data S1 Raw Data

## Additional Information and Declarations

### Data Availability

The following information was supplied regarding data availability: The clinical raw data is available in the Supplemental File.
